# Chemical Coupled PEDOT:PSS/Si Electrode: Suppressed Electrolyte Consumption Enables Long-Term Stability

**DOI:** 10.1007/s40820-020-00564-5

**Published:** 2021-01-08

**Authors:** Xuejiao Liu, Zhixin Xu, Asma Iqbal, Ming Chen, Nazakat Ali, CheeTongJohn Low, Rongrong Qi, Jiantao Zai, Xuefeng Qian

**Affiliations:** 1 School of Chemistry and Chemical Engineering and State Key Laboratory of Metal Matrix Composites, Shanghai Electrochemical Energy Devices Research Center, Shanghai Jiao Tong University, Shanghai, 200240 P. R. China; 2 Warwick Electrochemical Engineering Group, Energy Innovation Centre, WMG, University of Warwick, Coventry, CV4 7AL UK

**Keywords:** Conductive binder, PEDOT:PSS, Cross-link, XPS depth analysis, Stable SEI, Lithium-ion batteries

## Abstract

Huge volume changes of Si during lithiation/delithiation lead to regeneration of solid-electrolyte interphase (SEI) and consume electrolyte. In this article, γ-glycidoxypropyl trimethoxysilane (GOPS) was incorporated in Si/PEDOT:PSS electrodes to construct a flexible and conductive artificial SEI, effectively suppressing the consumption of electrolyte. The optimized electrode can maintain 1000 mAh g^−1^ for nearly 800 cycles under limited electrolyte compared with 40 cycles of the electrodes without GOPS. Also, the optimized electrode exhibits excellent rate capability. The use of GOPS greatly improves the interface compatibility between Si and PEDOT:PSS. XPS Ar^+^ etching depth analysis proved that the addition of GOPS is conducive to forming a more stable SEI. A full battery assembled with NCM 523 cathode delivers a high energy density of 520 Wh kg^−1^, offering good stability.

**Electronic supplementary material:**

The online version of this article (10.1007/s40820-020-00564-5) contains supplementary material, which is available to authorized users.

## Highlights

- γ-Glycidoxypropyl trimethoxysilane (GOPS) was incorporated in Si/PEDOT:PSS electrodes to construct a flexible and conductive artificial solid-electrolyte interphase (SEI).
- XPS Ar^+^ etching depth analysis proved that the addition of GOPS is conducive to forming a more stable SEI.
- A full battery assembled with NCM 523 cathode delivers a high energy density of 520 Wh kg^-1^, offering good stability.

**Electronic supplementary material:**

The online version of this article (10.1007/s40820-020-00564-5) contains supplementary material, which is available to authorized users.

## Graphical abstract

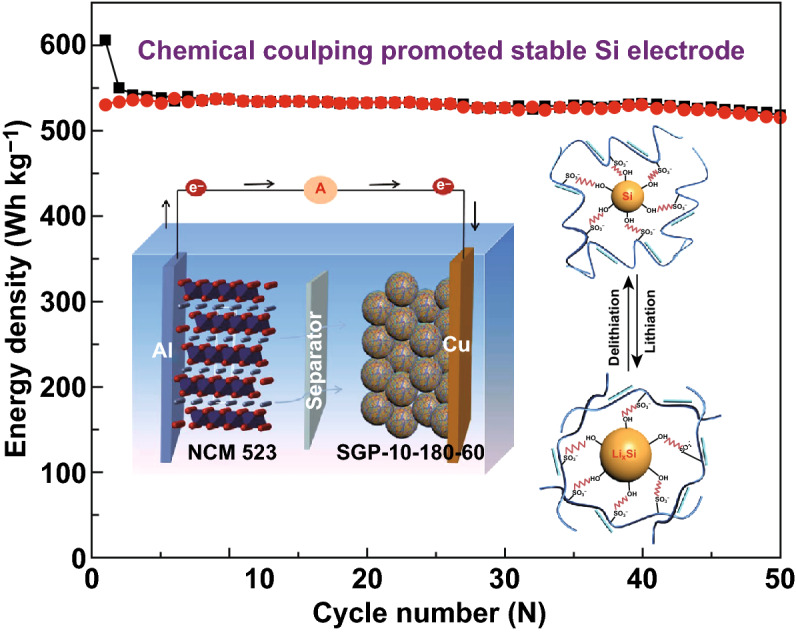

## Introduction

Si, as one of the most promising anode materials for lithium-ion batteries, has been investigated for decades due to its highest theoretical specific capacity (4200 mAh g^−1^) [1, 2]. However, Si suffers from huge volume changes (> 300%) during lithiation/delithiation, which causes active material pulverization and exfoliation from the collector. Both problems can now be relieved by nano-Si [3–5] and surface-bonded binders [6]. However, there will be a solid electrolyte interface (SEI) on the anode surface as the strong reducibility leads to the reduction and decomposition of the electrolyte. The formation of SEI helps to avoid direct contact between the active material and the electrolyte. But huge volume expansion of Si during lithiation also causes SEI rupture, so additional electrolyte and Li^+^ are irreversibly consumed repairing regenerations, which severely limits the Coulombic efficiency and cyclic stability. This problem is usually ignored in most anode studies due to excessive electrolyte in half-cell designs with Li metal as a counter electrode. To achieve high energy density in the actual full battery, the amount of electrolyte will be limited. However, SEI regeneration will overconsume electrolytes, resulting in sudden battery death. An ideal solution would be creating an artificial SEI on Si via an organic/polymer coating layer [7–10], which can effectively control the contact between electrolyte and active material. Nevertheless, the artificial SEI is usually insulating that cannot transfer both Li^+^ and electrons well. Thus, it is necessary to construct a flexible artificial SEI that is dual conductive for electronics and ions.

Conventional Si electrodes contain nearly 40 wt% inactive materials, including conductive additive and insulating binders, such as PAA [11], CMC, or alginate [12]. Conducting polymers with dual functions as a binder and conducting additives have been developed for Si anodes [13–16]. Among that, PEDOT is an excellent electronic conductor due to its rigid conjugated backbone [17, 18] and has been explored in lithium-ion batteries via *in situ* polymerization of EDOT [19–21]. However, poor ions diffusion ability and bad elasticity of pure conjugate rigid backbone may lead to poor cycling stability. Commercial PEDOT:PSS is a mixed ion-electron conductor. Besides, self-healing properties and improved elasticity of PEDOT:PSS make it more suitable as a binder for Si anodes [22, 23]. Previous reports focused on improving mechanical performance or electrical conductivity of PEDOT:PSS itself. Huo’s group did cross-link of D-sorbitol and vinyl acetate-acrylic onto PEDOT:PSS chains to form a highly stretchable conductive glue and used as a high-performance binder [23]. Zhou’s group assembled PEO and PEI onto PEDOT:PSS chains and used as a binder with high ion and electron conductivities [24]. Our previous work used multivalent ion-cross-linking PEDOT:PSS to enhance the strength by forming 3D structured polymers [25].

Although the above researches have alleviated the disadvantages of the Si negative electrode to a certain extent, these are mainly focused on optimizing the mechanical performance of binder but ignored the interface interaction with Si. The hydrophobicity and negativity of Si results in incompatible interfaces and charge repulsion, limiting the development of PEDOT:PSS-based binders. Therefore, improvements in interfacial properties may play a crucial role [26–28]. In another work, we improved interfacial compatibility and conductivity by glycerol cross-linking [12], but the interaction between Si and glycerol was relatively weak and needed to be further strengthened.

In this paper, considering the overall electrode structure, a silane coupling agent, γ-glycidoxypropyl trimethoxysilane (GOPS), was incorporated as a bridge between the Si and PEDOT:PSS to form a spatial network structure for the high-performance electrode (SGP electrode). The use of GOPS greatly improves the interface compatibility between active material and binder. The SGP-10-180-60 electrode exhibited higher peeling force, smaller *R*_SEI_ resistance after cycling. XPS Ar^+^ etching depth analysis proved that the addition of GOPS is conducive to forming a more stable SEI. As a result, the obtained SGP-10-180-60 electrode showed better cycling stability and rate capability. After cross-linking, the SGP-10-180-60 electrode could cycle validly for ~ 800 times (compared with 40 cycles for the electrodes before cross-linking) under a set specific capacity of 1000 mAh g^−1^. Full battery composed of SGP-10-180-60 anode with NCM 523 cathode exhibited a high capacity of ~1.5 mAh cm^−2^ and displayed ICE of 87.6%. The initial resultant specific energy is 530 Wh kg^−1^ (based on the total weight of anode and cathode), and after 60 cycles, it maintained 485 Wh kg^−1^.

## Experimental Section

### Materials

PEDOT:PSS (1.4 wt% in water) was obtained from Adamas, and the Si NPs were supplied by Jingxing alloy welding materials co. LTD. (γ-Glycidyloxypropyl) trimethoxysilane (GOPS) was obtained from Adamas-beta. Polyacrylic acid (PAA) was supplied by Alfa-Aesar. All the chemicals were used directly without further purification.

### Preparation of a Series of SGP Electrodes

In a typical experiment, a mixture of 80 mg of Si NPs, 20 mg (1.428 mL) of PEDOT:PSS, and 10 mg of GOPS was stirred to form an uniform slurry. After stirring for 6 h, the slurry was coated on a copper foil using a doctor blade method and dried overnight at 80 °C. Then, the as-prepared electrodes were further heat-treated at 180 °C for 60 min. The resulting electrode is denoted as SGP-10-180-60 electrode. For comparison, the electrodes with 5 mg GOPS and 20 mg GOPS were also prepared using the same method except for the mass of GOPS, and were denoted as SGP-5-180-60 and SGP-20-180-60, respectively. Similarly, the electrodes of SGP-10-160-60, SGP-10-200-60, SGP-10-180-0, SGP-10-180-10, and SGP-10-180-120 were also designed following same methodology. The Si-PEDOT:PSS electrode was prepared by the same method but without addition of GOPS cross-linker.

### Preparation of Si-PAA-SP Electrode

In a typical experiment, a mixture of 80 mg Si nanoparticles, 20 mg PAA binder, and 10 mg super P was stirred to form a uniform slurry. After stirring for 6 h, the slurry was coated on a copper foil using a doctor blade method and dried overnight at 80 °C.

### Preparation of SGP-10–180-60||NCM 523 Full Batteries

NCM 523 electrodes with a mass loading of 10 mg cm^−2^ were supplied by Ningbo Shanshan Co., Ltd. The SGP-10-180-60 anode and NCM 523 cathode had been activated by pre-cycling 3 times at low current (anode: 200 mA g^−1^, cathode: 20 mA g^−1^) before assembling into full battery.

### Characterizations

X-ray diffraction measurements were carried out on Shimadzu XRD-6000 using Cu Kα radiation (*λ* = 1.5418Ǻ), and 2*θ* from 10 to 80 ° with a scan rate of 6 ° min^−1^. TEM (PHILIPS, Tecnai 12) and FESEM (JEOL, JSM-6700F or FESEM, Hitachi, S-4800) were conducted to study the morphological features of the samples. Raman spectroscopy was conducted on a Jobin-Yvon LabRam HR80 spectrometer to examine the chemical composition using a 532 nm laser. Fourier transform infrared spectrometer (FTIR) spectra were obtained by Nicolet 6700 spectrometer. X-ray photoelectron spectroscopy (XPS) was performed on a VG Scientific ESCLAB 220 iXL X-ray photoelectron spectrometer. Peeling test was measured with a universal electromechanical tester (Instron 4465) to evaluate the binder strength, and an electrode sample prepared in 30 mm width and 80 mm length was attached to 3 M tape.

### Electrochemical Measurements

Electrochemical tests were conducted using CR2016 coin half cells. Electrochemical cells were assembled with as-prepared electrodes, metallic lithium foil as counter electrode. The electrolyte is 1 M solution of LiPF_6_ in a mixture of ethylene carbonate (EC)/diethylene carbonate (DEC) (1:1, vol%) with 10% fluoroethylene carbonate (FEC) was used as the additive and the separator is obtained from Celgard 2400. The weight of each piece of electrode is around 1.0 mg. The SGP-10-180-60 || NCM 523 full batteries were assembled by similar method. The half-cells were galvanostatic discharged/charged in the fixed voltage range of 0–1.5 V, and full cells were tested in 2.8–4.25 V by the battery testing system (LAND CT2001A model, Wuhan jinnuo Electronics, China). Cyclic voltammetry (CV) was implemented at 0.2 mV s^−1^ between 0.01–2.0 V. Electrochemical impedance spectroscopy (EIS) was performed on a CHI 650E electrochemical workstation in the frequency range from 100 kHz to 0.1 Hz.

## Results and Discussion

### Electrochemical Performance of Different Electrodes

**Fig. 1:**
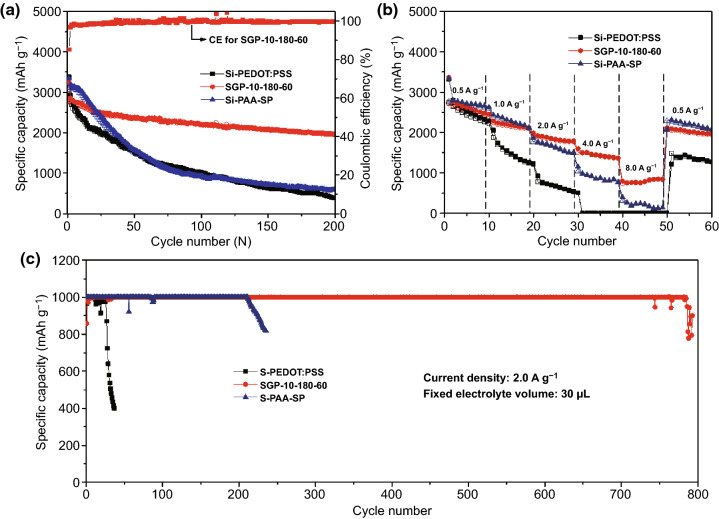
**a** Galvanostatic cycling test of Si-PEDOT:PSS electrode, SGP-10-180-60 electrode and Si-PAA-SP anode at current density of 1.0 A g^−1^. **b** Rate capabilities of Si-PEDOT:PSS, SGP-10-180-60, and Si-PAA-SP anodes in current density of 0.5–8.0 A g^−1^. **c** Si-PEDOT:PSS, SGP-10-180-60, and Si-PAA-SP electrodes tested with quantitative lean electrolyte of 30 μL and a fixed Li insertion capacity of 1000 mAh g^−1^ at 2.0 A g^−1^

In this work, commercial Si nanoparticles were used as active material without further purification (Fig. S1). To investigate the effects of different cross-linking conditions on cyclic stability, the galvanostatic cycling tests of SGP anodes at a diverse cross-linking time (Fig. S2a), different cross-linking temperatures (Fig. S2b), and different GOPS amounts (Fig. S2c) were conducted at 1.0 A g^−1^. It is evident from Fig. S2 that the best cyclic stability is obtained when the cross-linking process was kept 60 min at 180 °C, and 10 mg GOPS added. After 200 cycles, the SGP-10-180-60 electrode can maintain a high reversible specific capacity of 1957.6 mAh g^−1^, the capacity retention is 70.8%. To better illustrate the cyclic stability of the SGP-10-180-60 electrode, the electrode used PAA as a binder was prepared with the same ratio (where the GOPS was replaced with super P) and marked as Si-PAA-SP electrode. After 200 cycles, the specific capacity and capacity retention values were 584.6 mAh g^−1^ and 19.1%, lower than the SGP-10-180-60 electrode (Fig. 1a). For comparison, the electrode with pure PEDOT:PSS as a binder (Si-PEDOT:PSS electrode) was also prepared. After 200 cycles, the specific capacity and capacity retention were 384.1 mAh g^−1^ and 13.6%. What is more, it is clear that the Coulombic efficiency of SGP-10-180-60 and Si-PEDOT:PSS electrodes is better than the Si-PAA-SP electrode (Fig. S3). This could be inferred by the dense structure of conductive polymer reducing the electrolyte permeation and the direct contact between electrolyte and Si. The rate capabilities were tested in the range of 0.5–8.0 A g^−1^ of SGP-10-180-60, Si-PEDOT:PSS, and Si-PAA-SP anodes (Fig. 1b). The SGP-10-180-60 showed the best rate capability, and the specific capacity could maintain ~ 760 mAh g^−1^ at 8.0 A g^−1^, owing to the high electrical conductivity of PEDOT:PSS. The electrodes before and after cross-linking were tested with a quantitative lean electrolyte of 30 μL and a fixed Li insertion capacity of 1000 mAh g^−1^ at 2.0 A g^−1^ as shown in Fig. 1c. The electrode after cross-linking (SGP-10-180-60) can maintain this capacity for nearly 800 times compared with Si-PEDOT:PSS electrode that can maintain for less than 40 cycles and the Si-PAA-SP electrode for 220 times. The reason for such noteworthy capacity may be that the SGP-10-180-60 electrode can form a stable SEI after cross-linking.

### Mechanism of the Cross-linking Reaction

**Fig. 2:**
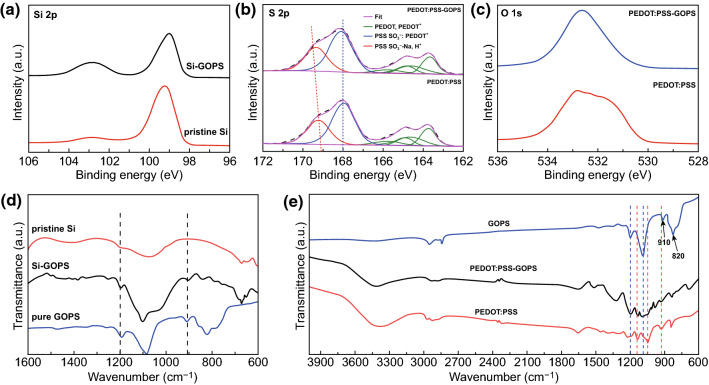
XPS analysis: **a** Si 2p region of raw Si and after treatment by GOPS; **b, c** S 2p and O 1s spectrum of pure PEDOT:PSS and after treatment by GOPS. FTIR measurements of **d** raw Si NPs, pure GOPS, and Si after treatment with GOPS; **e** pure PEDOT:PSS, pure GOPS, and PEDOT:PSS after treatment by GOPS

To gain insight into the cross-linking reaction mechanism among PEDOT:PSS, GOPS, and Si nanoparticles, a series of XPS were conducted. As shown in Fig. 2a, broad peaks within the range of 102–105 eV are evident in both measured samples ascribed to the amorphous SiO_x_ layer [29]. The conclusion agrees with the TEM results. In Fig. S1, the area ratio of oxidation state peaks has been increased after treatment with GOPS, indicating the interaction between Si and GOPS. Besides, the peaks centered at ~ 99 eV can be indexed to Si^0^, and there was a shift of ~ 0.3 eV toward high energy after treatment with GOPS. Figure 2b shows the S 2p spectra consisting of two major parts, one from PEDOT at low binding energy (163-166 eV) and the other from PSS at high binding energy (166–172 eV) [30, 31]. After treating with GOPS, the PSS region’s S 2p spectra relate to the -SO_3_^−^ groups interacting with Na^+^ or H^+^ (~ 169 eV) has a shift to higher binding energy, while the PSS interacting with PEDOT and PEDOT region stays constant. The XPS revealed the cross-linking between PEDOT:PSS and GOPS [32–34]. The O 1s spectra after treating with GOPS also showed a shift to high binding energy (Fig. 2c), suggesting the change in the chemical environment of the oxygen atoms after getting treated with GOPS.

FTIR measurement is conducted to confirm the cross-linking among Si, GOPS, and PEDOT:PSS binder. As shown in Fig. 2d, after treated with GOPS, two distinct peaks located at ~ 1200 and ~ 900 cm^−1^ were observed and assigned to -CH_2_ wagging, whereas the main band around 1100 cm^−1^ is due to Si-O-Si symmetric stretching mode [35], which is the strong evidence of cross-linking between Si and GOPS. From Fig. 2e, it can be seen that in the spectrum of the PEDOT:PSS after treatment with GOPS, there are two peaks centered at ~ 1200 and ~ 1080 cm^−1^ that also exist in GOPS and three peaks at 1130, 1000, and 920 cm^−1^ are consistent with pure PEDOT:PSS. As it can be seen that in pure GOPS spectra, there are two peaks at 820 and 910 cm^−1^, which can be assigned to epoxide moieties of GOPS [36]. After reaction with PEDOT:PSS, the two epoxide absorption bands disappeared, which further confirmed the reaction between GOPS and PEDOT:PSS. From the above analysis, GOPS acts as a bridge connecting Si with PEDOT:PSS. This structure is conducive to the contact between Si and binder and further facilitates a dense SEI film formation. Raman spectra of both pure PEDOT:PSS and after treatment by GOPS show the typical features of PEDOT:PSS [37]. There are no obvious Raman shifts of PEDOT:PSS after treatment with GOPS (Fig. S5), indicating that GOPS incorporation has not changed the main chain structure of PEDOT:PSS.

**Fig. 3:**
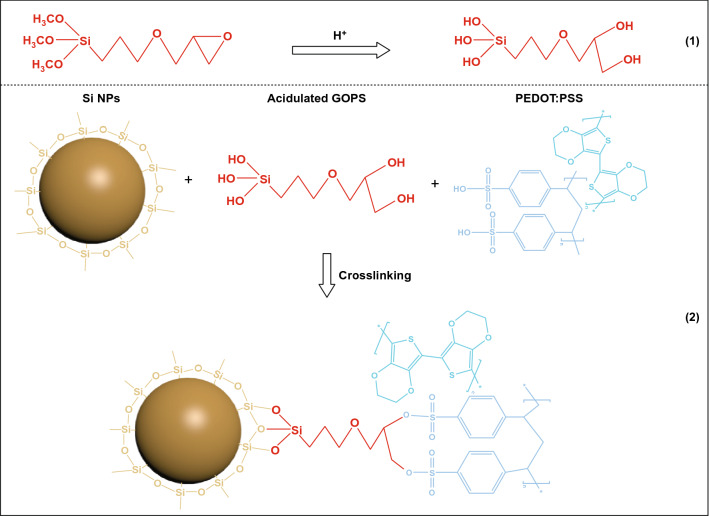
Schematic diagram of the cross-linking interactions: (1) GOPS under acidic conditions; (2) cross-linking among Si nanoparticles, GOPS, and GOPS

Based on the above characterizations, the cross-linking reactions between PEDOT:PSS, GOPS, and Si are shown in Fig. 3. The addition of (γ-glycidyloxypropyl) trimethoxysilane (GOPS) in acidic PEDOT:PSS solution (1.4 wt% in water, pH = 2) causes the epoxy groups to be opened to form two hydroxyl groups (–OH) (chemical Eq. 1); these hydroxyl groups (–OH) interact strongly with –OSO_3_H in PSS chains by hydrogen bonding as shown in chemical Eq. 2 [30]. The methoxyl groups (–OCH_3_) on the other end of GOPS will be hydrolyzed, and the more stable silanol (Si–OH) groups are generated [38]. From Fig. S1, we can find that there is a layer of amorphous SiO_x_ on Si nanoparticles, Si–OH groups of hydrolyzed GOPS and surface of Si nanoparticles can react with each other releasing water molecules. In brief, GOPS finally realized the cross-linking of Si active material and binder PEDOT:PSS to form a spatial network structure, to increase the strength of the adhesive and the contact area between Si and PEDOT:PSS.

### Characterization of Different Electrodes After Cycling

**Fig. 4:**
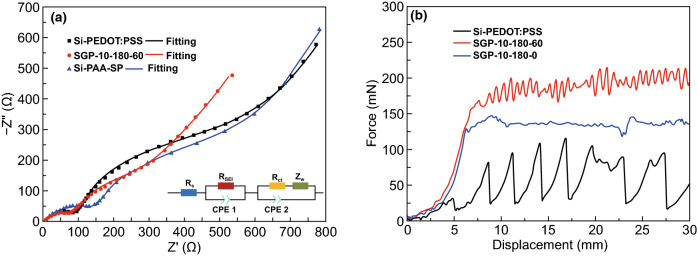
**a** Nyquist plots of SGP-10–180-60, Si-PEDOT:PSS, and Si-PAA-SP anodes after 200 cycles at 1.0 A g^-1^. **b** Peeling tests of the different electrodes. F-D relation of SGP-10-180-0, SGP-10-180-60, and Si-PEDOT:PSS anodes

The impedance spectroscopy results of SGP-10-180-60, Si-PEDOT:PSS, and Si-PAA-SP anodes after cycling 200 times at 1.0 A g^−1^ are shown in Fig. 4a (inset image is the corresponding fitting circuit model). The fitting resistances based on fitting equivalent are shown in Table S1. The EIS curves are composed of two semicircles (corresponding to *R*_SEI_ and *R*_ct_ resistances) in the medium-to-high frequency region and Warburg impedance (Z_w_). The SGP-10-180-60 electrode exhibited the smallest *R*_s_ and *R*_SEI_ resistances. To investigate electrodes’ mechanical properties before and after cross-linking with GOPS, peeling tests were conducted as shown in Fig. 4b. According to the F-D curves, the peeling force of Si-PEDOT:PSS is ~ 60 mN. After the addition of GOPS, the SGP-10-180-0 revealed a peeling force of ~ 125 mN. After being sufficiently cross-linked with GOPS, the SGP-10-180-60 presented the highest peeling force value of ~ 200 mN, indicating that GOPS cross-linking also encourages the improvement in the mechanical properties of the electrodes.

**Fig. 5:**
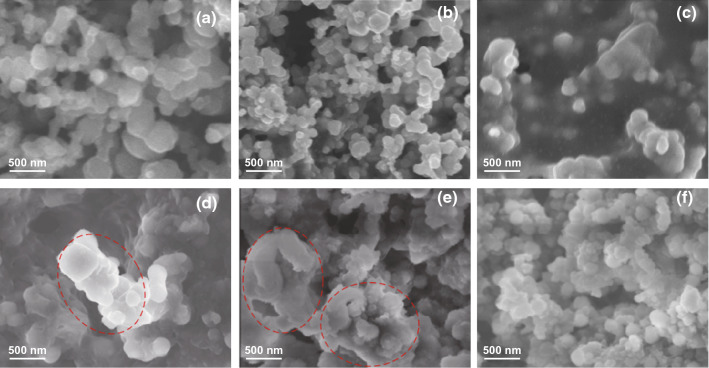
SEM images of Si-PAA-SP (**a, d),** Si-PEDOT:PSS **(b, e),** and SGP-10-180-60 (**c, f).** Anodes before cycling (**a–c)** and after cycling 200 times at 1.0 A g^−1^ (**d–f)**

To better understand the cross-linked electrodes’ improved cycling stability, the SEM was conducted for the Si-PAA-SP, Si-PEDOT:PSS, and SGP-10-180-60 electrodes before cycling and after 200 cycles at 1.0 A g^−1^ as shown in Fig. 5. It can be observed that Si nanoparticles are uniformly distributed on the Si-PAA-SP electrode and Si-PEDOT:PSS electrode (Fig. 5a, b). In comparison, there is a layer of polymer on the SGP-10-180-60 electrode (Fig. 5c). After 200 cycles at 1.0 A g^−1^, evident morphology changes are noticed on Si-PAA-SP and Si-PEDOT:PSS electrodes as marked by red circles in Fig. 5d, e, considered as pulverization of Si particles [39]. By contrast, no obvious detectable morphology change of Si nanoparticles was found for the SGP-10-180-60 electrode after 200 cycles (Fig. 5f).

**Fig. 6:**
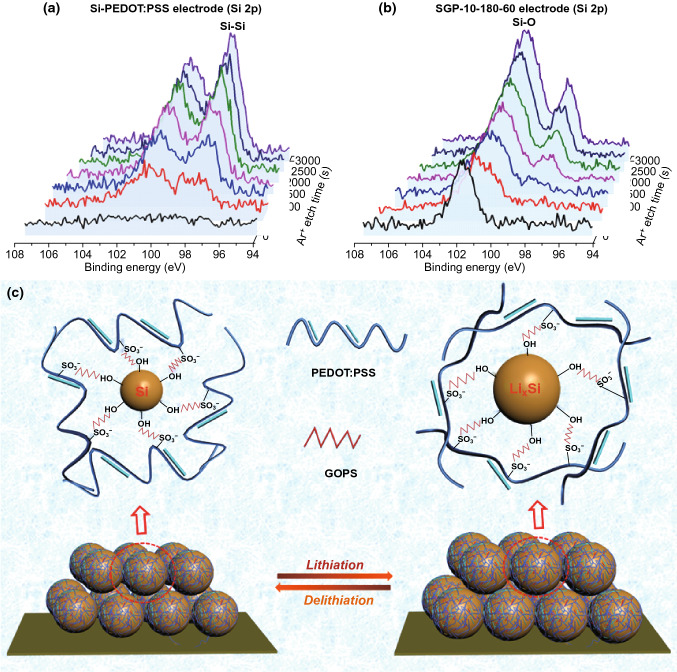
Si 2p spectra of XPS Ar^+^ etching depth analysis of **a** Si-PEDOT:PSS and **b** SGP-10-180-60 electrodes after 100 cycles at 2.0 A g^−1^. **c** Schematic illustration of the proposed mechanism explaining the electrochemical performance of SGP-10-180-60 anodes

Figure 6 presents XPS Ar^+^ etching depth analysis of Si-PEDOT:PSS and SGP-10-180-60 electrodes after 100 cycles with a quantitative lean electrolyte (30 μL) and a fixed capacity of 1000 mAh g^−1^ at 2.0 A g^−1^ and the corresponding SEM images are shown in Fig. S6. The etching procedure included 6 steps, with an etching time of 500 s per step. Given that, the etch depth of Ar^+^ is about 1.3 nm per 100 s. Each etch step corresponded to ~ 6.5 nm. From Fig. 6a, b, the different behavior of two electrodes surfaces is obvious (etching time is 0 s). The Si-PEDOT:PSS electrode showed no Si signal when the SGP-10-180-60 electrode displayed only the oxidation state of Si signal. It could be inferred that the surface of the electrodes after the cycling formed SEI. For the Si-PEDOT:PSS electrode, the SEI contained no Si element, while for the SGP-10-180-60 electrode, the Si of oxidized state (102–104 eV) participated in the formation of the SEI. As can be deduced from the above analysis, the main state of raw Si NPs is Si^0^ (~ 99 eV). Therefore, the Si of oxidized state on the surface of the electrode after cycling comes from GOPS. After etched one step (500 s), the Si signal was detected on the surface of the Si-PEDOT:PSS electrode, which was also confirmed by SEM images (Fig. S6a, b). After cycling, the Si-PEDOT:PSS electrode showed obvious cracks (~ 3.7 μm), revealing that the SEI had been destroyed and generated repeatedly, so the signal of active material Si was detected by etching only one step. As for the SGP-10-180-60 electrode, it can be seen from the SEM images (Fig. S6c, d) that the surface of the electrode after cycling is relatively uniform i.e., without obvious cracks. The signals of active material Si were detectable from the third steps of etching (1000 s). The XPS Ar^+^ etching depth analysis peak fitting and relevant quantitative analysis of Si–Si and Si–O were added in Figs. S7, S8 and Table S2. With the increase in etching time, the Si–Si bond content of Si-PEDOT:PSS electrode increased from 27 to 40%, and after the etching 1500 s, the content remained at 40%. While the Si–Si content of the SGP-10-180-60 electrode gradually increased from 0 to 20% with the etching time increase from 0 to 3000 s. It can be inferred that the addition of GOPS favors and contributes to forming a more stable SEI, which can alleviate the problems of SEI being destructed and repeated generation.

Overall, the mechanism is shown in Fig. 6c, the superior performance of SGP-10-180-60 electrodes can be attributed to the favorable properties of GOPS: (1) high conductivity and good film-forming properties of PEDOT:PSS are conducive to form a desired SEI on the Si surface; (2) GOPS as a typical cross-inker [30] can improve the stability, elasticity [40], mechanical stability [41], and adhesion [42] of PEDOT:PSS; therefore, the incorporation of GOPS can better alleviate the volume expansion of Si; (3) the interaction between the GOPS and Si improved the stability of Si electrode and made the Si not easy to fall off. (4) GOPS cross-linked PEDOT:PSS is conducive to the formation of a more stable SEI, which can suppress the continued consumption of electrolyte.

### Characterization of Full Batteries

**Fig. 7:**
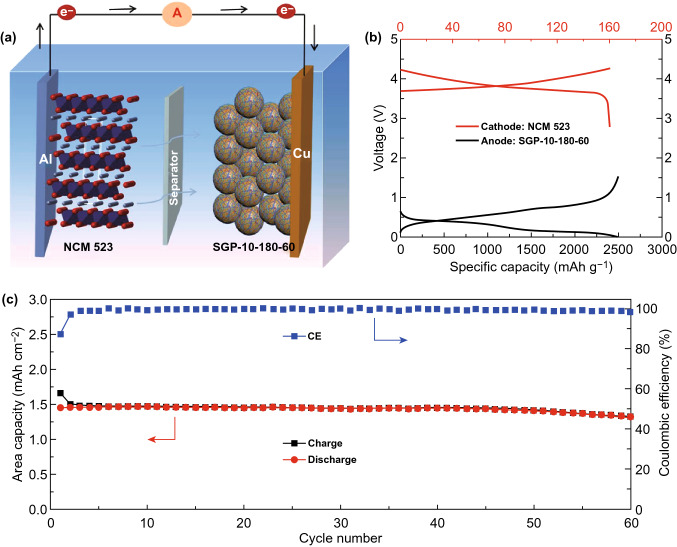
**a** Diagram of full battery assembly, the mass loading (10 mg cm^−2^) of an NCM 523 cathode vs. a capacity balanced SGP-10-180-60 anode. **b** Typical cathode/anode cycling plots. **c** Cycling performance of full-cell at 20 mA g^−1^ with prelithiated SGP-10-180-60 anode and a NCM 523 cathode

To further explore the practicability of the SGP-10-180-60 electrode, a full battery by a commercial Li-rich cathode was tested (NCM 523, SEM images of NCM 523 electrodes are shown in Fig. S9), as shown in Fig. 7a. The reversible capacity of NCM 523 cathode was ~ 160 mAh g^−1^ at 2.8–4.25 V (Fig. S10); the mass loading employed was 10 mg cm^−2^, which is comparable to the cathode in practical mass loading of 15 mg cm^−2^ [43]. To balance this capacity, the SGP-10-180-60 anode had an active mass loading of only 0.8 mg cm^−2^. Figure 7b overlays the potential curves of SGP-10-180-60 and NCM 523 half-cells. Figure 7c shows the cycling performance of full battery at 20 mA g^−1^; the full battery delivers a high specific capacity of ~1.5 mAh cm^−2^ and exhibits a high ICE of 87.6% (after the initial three cycles, the Coulombic efficiency kept over 99%). The full cell presented an average discharge voltage of ~3.5 V. The initial resultant specific energy was 530 Wh kg^−1^. After 60 cycles, it maintained 485 Wh kg^−1^, based on the total weight of anode and cathode.

## Conclusions

In summary, we have designed a GOPS cross-linked PEDOT:PSS binder for Si electrodes. The cross-linked binder enhanced the contact between the Si and the binder and alleviated the volume expansion and contributed to the formation of a more stable and robust SEI to reduce the absorption of the electrolyte. The obtained SGP-10-180-60 electrode can maintain a high specific capacity of 1957.6 mAh g^−1^ after cycling 200 times, with a high capacity retention of 70.8%. What is more, the SGP-10-180-60 electrodes maintained up to ~ 800 times when tested with a quantitative lean electrolyte of 30 μL and a fixed Li insertion capacity of 1000 mAh g^−1^ at 2.0 A g^−1^. SGP-10-180-60 anode║NCM 523 cathode full battery exhibited an area capacity of ~1.5 mAh cm^−2^ and exhibited a high ICE of 87.6%. The initial resultant specific energy was 530 Wh kg^−1^, and after 60 times, it maintained 485 Wh kg^−1^, based on the total weight of anode and cathode. This study paves a new way of designing binders used in other high-capacity electrodes with a strong volume effect.

## Electronic supplementary material

Below is the link to the electronic supplementary material.

Supplementary file 1 (PDF 968kb)
