# Supplementary material for: Chemical Coupled PEDOT:PSS/Si Electrode: Suppressed Electrolyte Consumption Enables Long-Term Stability
Source: Nanomicro Lett. 2021 Jan 8;13:54. doi: 10.1007/s40820-020-00564-5 (PMC8187542; doi:10.1007/s40820-020-00564-5)
Supplement: Supplementary file 1 — (PDF 968kb) [file 40820_2020_564_MOESM1_ESM.pdf]

Supporting Information for

Chemical Coupled PEDOT: PSS/Si Electrode: Suppressed Electrolyte Consumption Enables Long Term Stability

Xuejiao Liu[1], Zhixin Xu[1], Asma Iqbal[1], Ming Chen[1], Nazakat Ali[1], CheeTongJohn Low[2], Rongrong Qi[1,]*, Jiantao Zai [1,]*, Xuefeng Qian[1,]*

[1]Shanghai Electrochemical Energy Devices Research Center, School of Chemistry and Chemical Engineering and State Key Laboratory of Metal Matrix Composites, Shanghai Jiao Tong University, Shanghai, 200240, P. R. China

[2]Warwick Electrochemical Engineering Group, WMG, Energy Innovation Centre, University of Warwick, CV4 7AL, United Kingdom

*Corresponding authors. E-mail: rrqi@sjtu.edu.cn (Rongrong Qi); zaijiantao@sjtu.edu.cn (Jiantao Zai); xfqian@sjtu.edu.cn (Xuefeng Qian)

Supplementary Tables and Figures

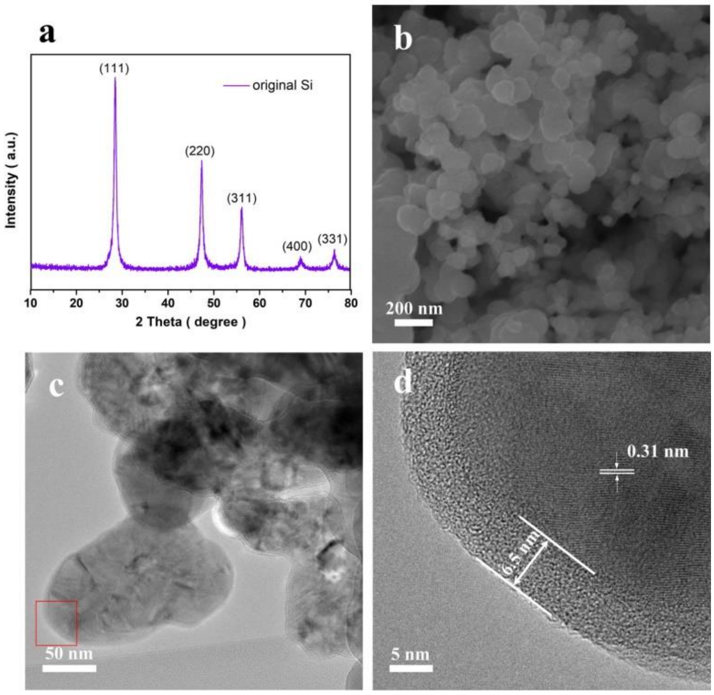

Fig. S1 Structure of the nano Si: **a** XRD pattern **b** SEM image **c** TEM image and HRTEM image of pristine Si nanoparticles. The HRTEM image indicate that the particles are crystalline with a distinguishable oxide layer with thickness of 6.5 nm.

XRD patterns in Fig. S1 show that all the peaks can be indexed to cubic crystalline Si (JCPDS No. 5-565). From SEM and TEM images, it can be found that the size of Si nanoparticles is about 80 nm. HRTEM image in Figure S1d indicates the interplanar distance of 0.31 nm which can be ascribed to the (111) plane of Si and a layer of amorphous SiO_2 was observed on Si nanoparticles.

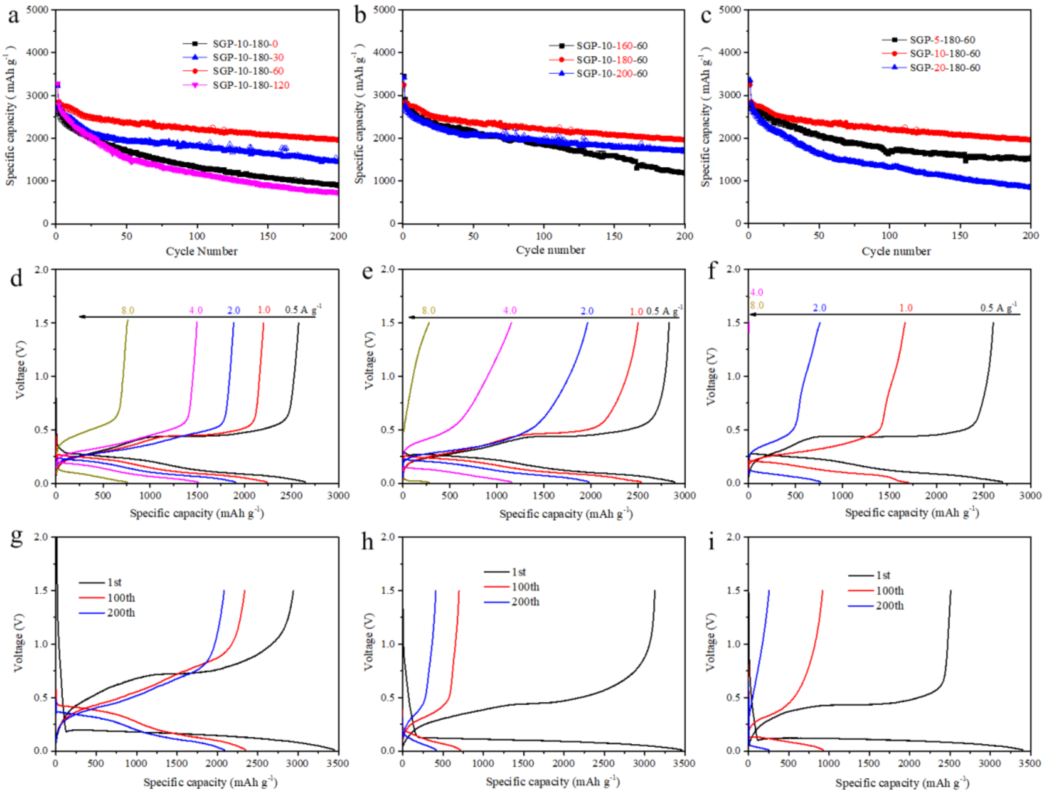

Fig. S2 a Galvanostatic charge and discharge test of electrodes of different crosslinking time from 0 to 120 min at 1.0 A g^{-1}. (Here, SGP stands for Si, GOPS and PEDOT: PSS electrode: the first number represents the amount of added GOPS (mg); the second number represents the crosslinking temperature (℃); and the third number represents the crosslinking time (min). For example, SGP-10-180-60 represents 10 mg GOP, and the electrode is crosslinked at 180 ℃ for 60 min.); **b** galvanostatic charge and discharge test of SGP electrodes of different crosslinking temperature at 1.0 A g^{-1}; **c** galvanostatic charge and discharge test of SGP electrodes of different addition amount of GOPS at 1.0 A g^{-1}. Voltage profiles of SGP-10-180-60 **d,** Si-PAA-SP **e**, and Si-PEDOT:PSS **f** electrodes at different current densities. Voltage profiles of SGP-10-180-60 **g** Si-PAA-SP **h** and Si-PEDOT:PSS **i** electrodes at 1.0 A g^{-1}

From Fig. S2a, the best cyclic stability is obtained when the crosslinking process was kept 60 min at 180°C and 10 mg GOPS added. After 200 cycles, the SGP-10-180-60 electrode maintained a high reversible specific capacity of 1957.6 mAh g^{-1}, the capacity retention is 70.8% compared with 889.7 mAh g^{-1} and 33.8% of SGP-10-180-0 electrode, 1453.1 mAh g^{-1} and 54.1% of SGP-10-180-10 electrode, 732.5 mAh g^{-1} and 27.2% of SGP-10-180-120 electrode. Subsequently, the crosslinking time was fixed to 60 min to explore the effect of different crosslinking temperature as shown in

Fig. S2b. The reversible specific capacities and capacity retention are 1183 mAh g^{-1} and 42.9% of SGP-10-160-60 electrode. When the crosslinking temperature was increased to 200 °C after 200 cycles, 1685.4 mAh g^{-1} and 63.5% were remained, which is comparable to the SGP-10-180-60 electrode. Finally, the amounts of crosslinking agent have been investigated (Fig. S2c). When the adding amount of GOPS was changed to 5 and 20 mg, the specific capacities and capacity retention were 1511.4 mAh g^{-1} (54.4%) and 841.2 mAh g^{-1} (31.2%) after 200 cycles, respectively. The reason may be that 5 mg GOPS is not enough to fully cross-link PEDOT:PSS. It has been reported that the cross-linking procedure will reduce the ionic intrinsic conductivity of PEDOT:PSS, which may be the reason why the excessive addition of GOPS (20 mg) leads to the decreased cycle stability.

As shown in Fig. S2d-f, these three electrodes have similar discharge platforms at ~0.1 V and charge sloping plateaus from 0.1 to 0.6 V, corresponding typical lithiation and delithiation reactions. Among these electrodes, the SGP-10-180-60 electrode exhibits best rate capability, even at high current density of 8.0 A g^{-1}, the SGP-10-180-60 electrode shows high reversible capacity of ~760 mAh g^{-1}.

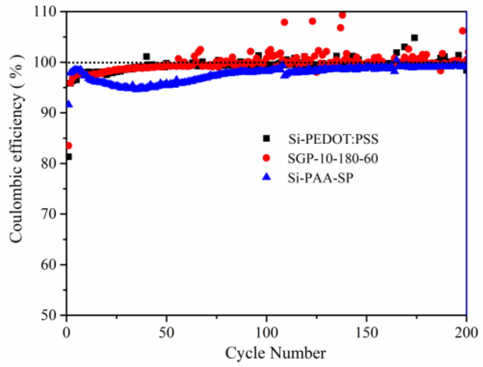

Fig. S3 Coulombic efficiency of Si-PEDOT:PSS electrode, SGP-10-180-60, electrode and Si-PAA-SP electrode at 1.0 A g^{-1}

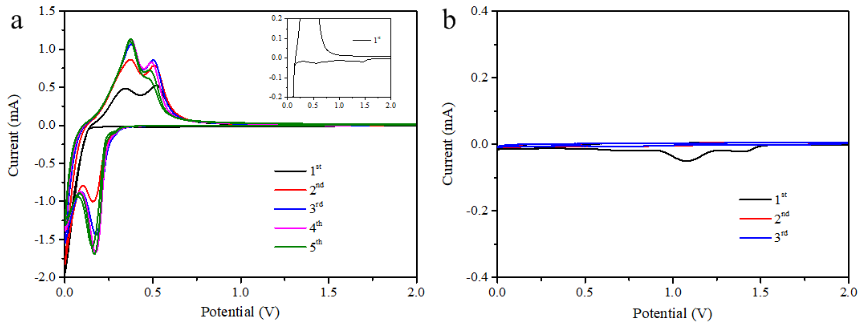

Fig. S4 a Cyclic voltammograms of SGP-10-180-60 electrode at scan rate of 0.1 mV s^{-1}; and **b** SGP-10-180-60 binder at scan rate of 0.1 mV s^{-1}

The electrochemical process of electrodes was investigated. Cyclic voltammograms of SGP-10-180-60 electrode at scan rate of 0.1 mV s^{-1} over the potential range of 0.01 - 2.0 V versus Li/Li^+ are shown in Fig. S4a. The SGP-10-180-60 electrodes show typical lithiation and delithiation peaks of Si. In the first cathodic scan, two peaks at ~ 1.1-1.4 V are observed, which are assigned to polymer binder, as shown in Fig. S4b. In addition, one peak at ~ 0 V is observed in the first cathodic scan, corresponding to conversion of Si to an amorphous Li_xSi phase. The peaks at 0.35 and 0.52 V in the anodic process could be ascribed to the conversion of Li_xSi to Si.

Table S1 Fitting resistances based on fitting equivalent

Electrodes	R_S	R_{SEI}	R_{ct}
**SGP-10-180-60**	5.1	83.2	258.9
**Si-PEDOT:PSS**	5.9	111.0	104.4
**Si-PAA-SP**	6.9	171.1	22.6

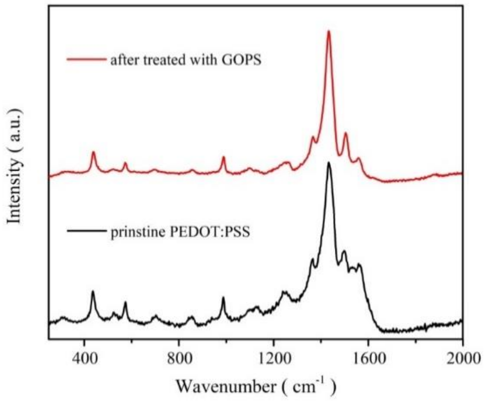

Fig. S5 Raman spectra for pristine PEDOT:PSS and after treatment with GOPS. Two samples show a characteristic PEDOT:PSS signal with no change discernible change in peak position or relative intensities seen for different samples.

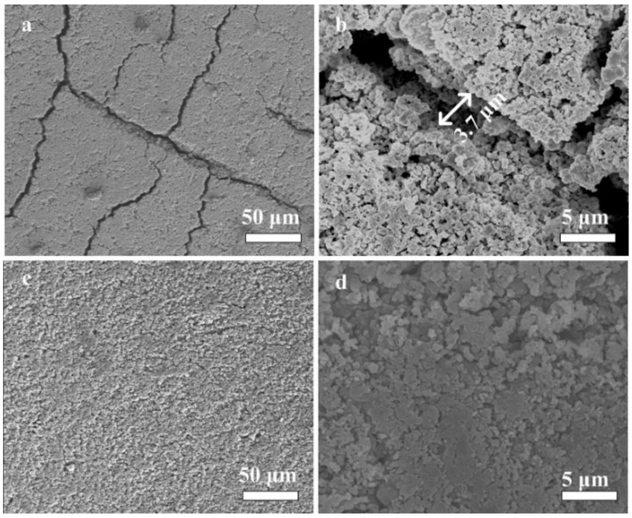

Fig. S6 SEM images of different electrodes after 100 cycles at 2.0 A g^{-1} before Ar^+ etching: **a, b** Si-PEDOT:PSS; **c, d** SGP-10-180-60

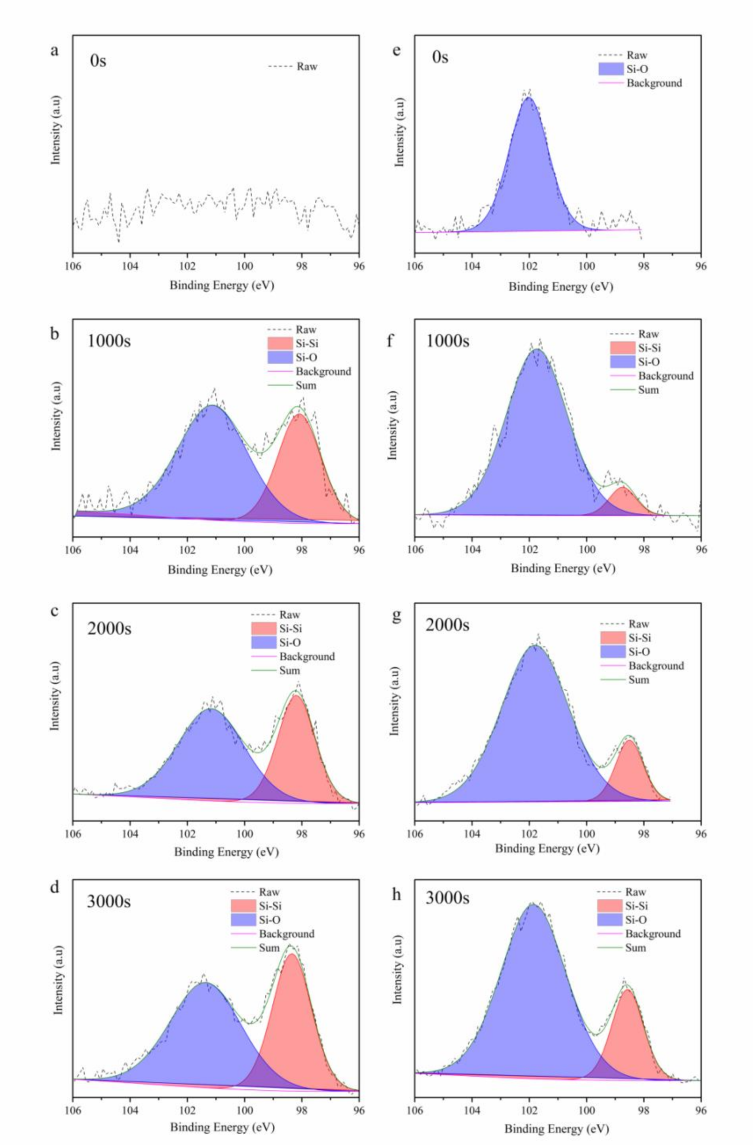

Fig. S7 Si 2p spectra of XPS Ar^+ etching depth analysis peak fitting of Si-PEDOT:PSS **a-d** and SGP-10-180-60 **e-h** electrodes

Table S2 Quantitative analysis of Si 2p XPS peak fitting

Ar^+ etch time (s)	Si-PEDOT:PSS electrode		SGP-10-180-60 electrode	
	Si-Si (%)	Si-O (%)	Si-Si (%)	Si-O (%)
0s	-	-	~0	~100
500s	27.0	73.0	4.8	95.2
1000s	37.0	63.0	6.9	93.1
1500s	41.0	59.0	11.9	88.1
2000s	40.0	60.0	13.7	86.3
2500s	42.8	57.2	18.2	81.7
3000s	41.4	58.6	20.0	80.0

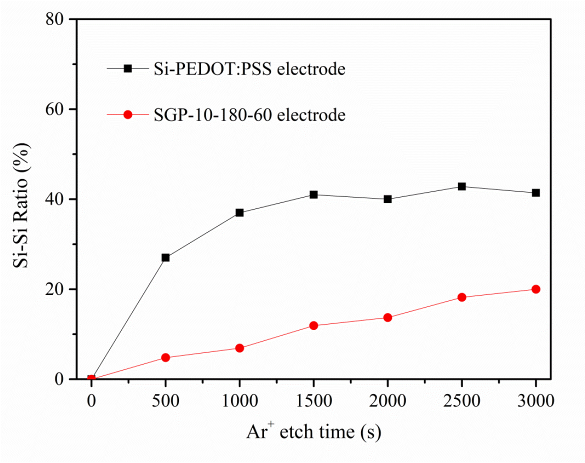

Fig. S8 Changes of Si-Si/Si-O ratios with Ar^+ etch time

The XPS Ar^+ etching depth analysis peak fitting and relevant quantitative analysis of Si-Si and Si-O were added in Fig. S8. With the increase of etching time, the Si-Si bond content of Si-PEDOT:PSS electrode increased from 27% to 40%, and after etching 1500 s, the content remained at 40%. While the Si-Si content of SGP-10-180-60 electrode gradually increased from 0% to 20% with the etching time increase from 0 s to 3000 s.

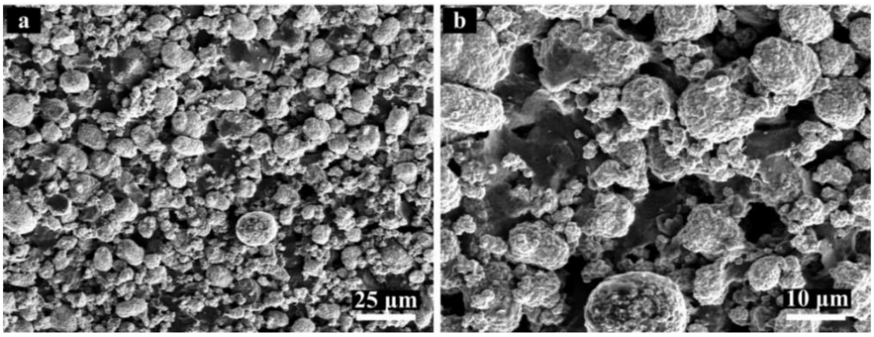

Fig. S9 SEM images of NCM 523 electrodes

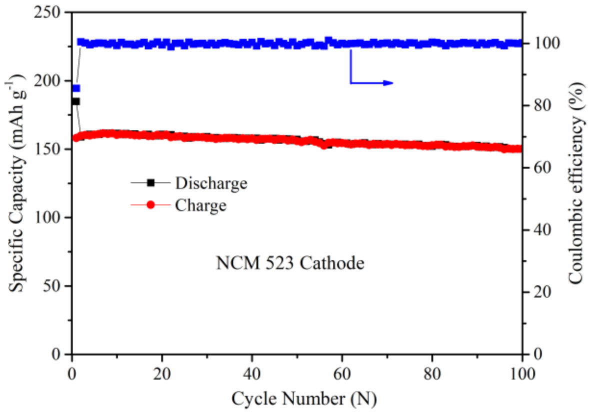

Fig. S10 Cycling performance of NCM 523 cathode half-cell at 60 mA g^{-1}

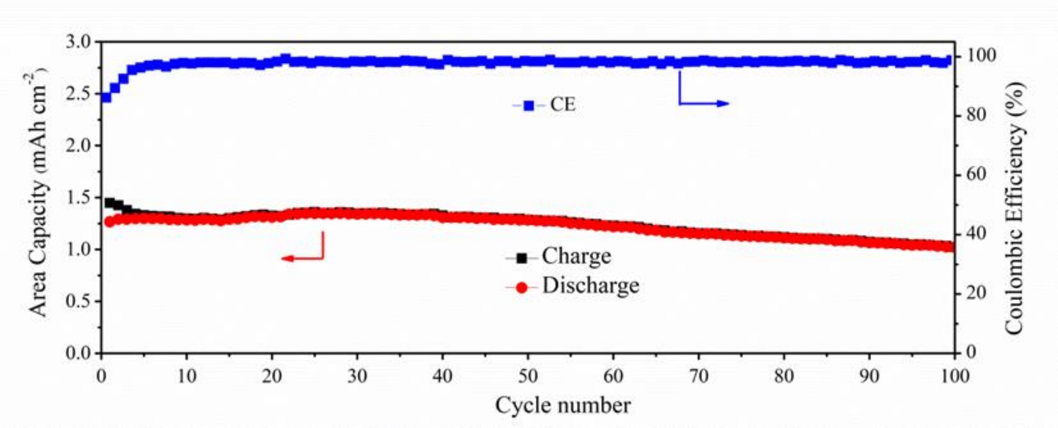

Fig. S11 Cycling performance of SGP-10-180-60 anode||NCM 523 cathode full-cell at 20 mA g^{-1}
